# Quantification of dental prostheses on cone‐beam CT images by the Taguchi method

**DOI:** 10.1120/jacmp.v17i1.5826

**Published:** 2016-01-08

**Authors:** Rong‐Fu Kuo, Kwang‐Ming Fang, Wong TY, Chia Yu Hu

**Affiliations:** ^1^ Medical Device Innovation Center, National Cheng Kung University Tainan Taiwan; ^2^ Department of Oral and Maxillofacial Surgery National Cheng Kung University Tainan Taiwan

**Keywords:** dental cone‐beam computed tomography (CBCT), prostheses, implant‐supported, gray value differences, Taguchi method

## Abstract

The gray values accuracy of dental cone‐beam computed tomography (CBCT) is affected by dental metal prostheses. The distortion of dental CBCT gray values could lead to inaccuracies of orthodontic and implant treatment. The aim of this study was to quantify the effect of scanning parameters and dental metal prostheses on the accuracy of dental cone‐beam computed tomography (CBCT) gray values using the Taguchi method. Eight dental model casts of an upper jaw including prostheses, and a ninth prosthesis‐free dental model cast, were scanned by two dental CBCT devices. The mean gray value of the selected circular regions of interest (ROIs) were measured using dental CBCT images of eight dental model casts and were compared with those measured from CBCT images of the prosthesis‐free dental model cast. For each image set, four consecutive slices of gingiva were selected. The seven factors (CBCTs, occlusal plane canting, implant connection, prosthesis position, coping material, coping thickness, and types of dental restoration) were used to evaluate scanning parameter and dental prostheses effects. Statistical methods of signal to noise ratio (S/N) and analysis of variance (ANOVA) with 95% confidence were applied to quantify the effects of scanning parameters and dental prostheses on dental CBCT gray values accuracy. For ROIs surrounding dental prostheses, the accuracy of CBCT gray values were affected primarily by implant connection (42%), followed by type of restoration (29%), prostheses position (19%), coping material (4%), and coping thickness (4%). For a single crown prosthesis (without support of implants) placed in dental model casts, gray value differences for ROIs 1–9 were below 12% and gray value differences for ROIs 13–18 away from prostheses were below 10%. We found the gray value differences set to be between 7% and 8% for regions next to a single implant‐supported titanium prosthesis, and between 46% and 59% for regions between double implant‐supported, nickel‐chromium alloys (Ni‐Cr) prostheses. Quantification of the effect of prostheses and scanning parameters on dental CBCT gray values was assessed.

PACS numbers: 87.59.bd, 87.57Q

## I. INTRODUCTION

Dental cone‐beam computed tomography (CBCT) systems are widely used in imaging of the oral and maxillofacial regions.^1^, ^2^, ^3^ A scanner rotates around the patient's head, obtaining up to nearly 300 to 600 distinct images at different angles. X‐ray attenuation of CBCT acquisition systems currently produces different CBCT gray values for bony and soft tissue structures in different areas of the scanned volume. Metal artifacts^(4)^ from dental metal prostheses are commonly observed in dental CBCT images.^5^, ^6^ Metallic restorations, crowns, and implants affect image quality due to beam hardening and streaks. Artifacts degrade image quality^(7)^ and result in a variety of dental CBCT gray values. Investigation of the quality and accuracy of dental CBCT in the imaging of dental structures was proposed in Holberg et al.^(7)^ and the quantification of image quality was analyzed by experienced observers. Comparison of several CBCT systems for image quality by score‐quantifying was proposed by Alqerban et al.^(8)^ These two researches use observers to evaluate image quality and focus on comparison of image quality obtained by different CBCT systems. Quantification of metal artifacts from several CBCT devices by calculating standard deviation of gray values of regions of interest (ROIs) was discussed in the work by Pauweis et al.,^(9)^ but there was only a range of artifact value affected by Titanium (Ti) and lead was proposed. Effect of metal artifacts on gray values surrounding dental implants using CBCT images was discussed in Naitoh et al.^(10)^ and mean pixel values between dental implants and neighboring teeth were discussed. The effects of artifacts on the assessment of finite element models for dental implants were evaluated in the study by Zannoni and colleagues.^(11)^ How distortion of gray values affect the prediction of bone quality for ROIs was proposed in Homoika et al.^(12)^ However, these previous evaluation and discussion cannot provide a quantitative effect of metal prosthesis on the surrounding voxel values. There is no standardized experiment parameter available for comparison of gray values variations due to dental CBCT scanning parameters and metal prostheses. The dental CBCT gray value is an indicator for predicting bone quality for ROIs. Evaluating bone quality surrounding teeth by CBCT gray values during orthodontic and implant treatment were demonstrated.^3^, ^13^, ^14^, ^15^, ^16^, ^17^ Although the metal artifact reduction (MAR) algorithm can reduce metal artifacts and improve gray values accuracy, its effect on artifact is still limited.^9^, ^18^

The aim of this study was to quantify effect contributed from dental prostheses and scanning parameters on CBCT images using a Taguchi method^19^, ^20^, ^21^ involving eight experiments, nine dental model casts of upper jaw, and seven design factors. This quantification would serve as a comparison of different systems, restoration types of dental prosthesis, and locations of prosthesis. Range of gray values variation due to these factors was analyzed. Systematic analysis of prosthesis effects on dental CBCT images was first to examine and present by analysis of variance (ANOVA) with 95% confidence. In this research seven factors with two levels (CBCTs, occlusal plane canting, implant connection, prostheses position, coping material, coping thickness, and types of dental restoration) were designed to analyze metal artifacts on CBCT gray value differences. We find that appropriate orthogonal array is Taguchi $L_{8}$ orthogonal array,^21^, ^22^ which has seven columns corresponding to the number of factors and eight rows corresponding to the number of tests. The prosthesis‐free dental model cast and eight dental model casts, including one or several prostheses, were prepared using the Taguchi's $L_{8}$ orthogonal array. Gray values for ROIs based on dental CBCT images were calculated for signal to noise ratio (S/N ratio) and S/N ratio was adopted to identify the significance of seven factors. The nine upper jaw casts were scanned by two dental CBCTs. Detailed descriptions of the experiment are in the Material & Methods section. The analysis for significance of design factors are described in the Results & Discussion section.

## II. MATERIALS AND METHODS

### A. Materials

**Figure 1:**
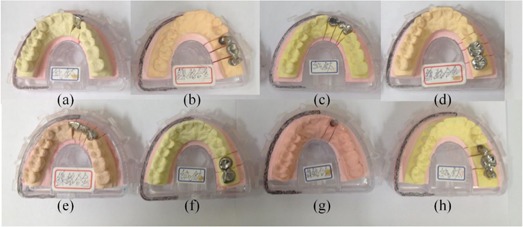
Eight dental casts ((a)–(h)) corresponding to experiment numbers 1–8 in Table 1. Casts (c), (d), (g), (h) are dental casts tilted by 15° during scanning. Casts (a) to (d) are scanned by DCT 100, and (e) to (h) are scanned by KaVo eXam.

In this work, the prostheses configurations placed in the dental cast model of upper jaw were according to an $L_{8}$ orthogonal array,^(21)^ as shown in Table 1. The orthogonal array comprises eight individual experiments, as indicated by eight rows. Eight simulations were required when the Taguchi method was employed. Numbers 1–8 in Table 1 correspond to dental model casts in Fig. 1(a) to (h). Seven independent variables were considered to evaluate the effect of scanning parameters and dental prostheses on dental CBCT gray values. Each variable have two set level values, see Table 2. The seven factors were: (A) CBCT scanners, (B) occlusal plane canting, (C) implant connection, (D) prostheses position, (E) coping material, (F) coping thickness, and (G) types of dental restoration. The prosthesis‐free images were acquired using a prosthesis‐free dental model cast. Array columns represent the seven factors (A–G), and entries in the array represent the level of these factors. Factor A with level 1 and level 2 means that the dental cast was scanned on a dental CBCT CareTech DCT 100 (Kaohsiung, Taiwan) and scanned on a CBCT KaVo eXam (Biberach, Germany). Factor B with level 2 indicates that the occlusal plane inclination is 15° (a dental model cast tilts down 15°) during scanning, see Table 2.

**Table 1:** $L_{8}$ orthogonal arrays for experiments (number under each investigated factor indicates levels assigned to that factor).

				*Factors*			
*Exp No*.	*(A) CBCTs*	*(B) Occlusal Plane Canting*	*(C) Implant Connection*	*(D) Prostheses Position*	*(E) Coping Material*	*(F) Coping Thickness*	*(G) Types of Restoration*
1	1	1	1	1	1	1	1
2	1	1	1	2	2	2	2
3	1	2	2	1	1	2	2
4	1	2	2	2	2	1	1
5	2	1	2	1	2	1	2
6	2	1	2	2	1	2	1
7	2	2	1	1	2	2	1
8	2	2	1	2	1	1	2

**Table 2:** Seven factors of dental casts with their levels.

*Factors*	*Level 1*	*Level 2*
A CBCT scanner	DCT 100	KaVoeXam
B Occlusal plane canting	0° (parallel to floor base)	15°
C Implant connection	Implant‐supported prosthesis	Prosthesis without support of implant
	acm20207Inline Graphicacm20207	acm20207Inline Graphicacm20207
D Prosthesis position	Anterior region	Posterior region
E Coping material	Titanium (Ti)	Nickel‐chromium alloys (Ni‐Cr alloys)
F Coping thickness	3 mm	0.4 mm
G Types of dental restoration	Crown	Bridge

For example, the first experiment was conducted with each factor at level one. The prosthesis was an anterior implant‐supported crown made of Titanium (Ti) with a coping thickness of 3 mm and scanned on a dental CBCT DCT 100 (see Fig. 1(a)). The dental model casts were comprised of: (a) an anterior implant‐supported Ti crown with a 3 mm coping thickness, (b) a posterior implant‐supported, nickel‐chromium (Ni‐Cr) alloy with a 0.4 mm coping thickness, (c) an occlusal plane canting of 15° and an anterior Ti bridge with a 0.4 mm coping thickness, (d) an occlusal plane canting of 15° and a posterior Ni‐Cr crown with a coping thickness of 3 mm, (e) an anterior Ni‐Cr alloy bridge with a coping thickness of 3 mm, (f) a posterior Ti crown with a 0.4 mm coping thickness, (g) an occlusal plane canting of 15° and an anterior implant‐supported Ni‐Cr alloy crown with a 0.4 mm coping thickness, and (h) an occlusal plane canting of 15° and a posterior Ti bridge with a 3 mm coping thickness. Customized dental implants were Ti and had the exactly same size as commercially available dental implants (12 mm in length and 3 mm in diameter). Dental model casts (Figs. 1(a)–(d)) were scanned on a dental CBCT DCT 100 and dental model casts (Figs. 1(e)–(h)) were scanned on KaVo eXam. Each experiment was repeated twice. A custom alignment jig was used for the alignment of dental model casts in scanning, and all ROIs were geometrically coincided. Each scanner had its specification parameters. The optimized parameters of the DCT 100 CBCT scanner were 100 kVp, 51.46 mAs/slice, 15 cm×9 cm field of view (FOV), and 0.25 mm slice thickness. The KaVo CBCT scanner's parameters were 120 kVp, 37.07 mAs/slice, 16 cm×9 cm FOV, and 0.25 mm slice thickness. The software used to analyze the gray values from DICOM data was MATLAB (MathWorks, Natick, MA).

### B. Methods

**Figure 2:**
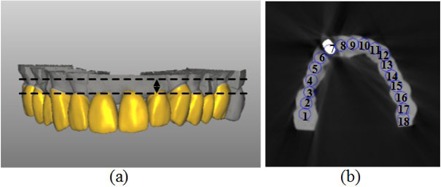
Four slices of a maxillary cast (a) were selected. A slice of the image sets of the 7th experiment (b) and the setting of the 18 ROIs for each slice in CBCT image sets.

For each image set, four consecutive slices of gingiva were selected (Fig. 2(a)). For our simulated dental casts, material of gingiva and teeth are all plaster, ROIs include part of gingiva that does not affect the quantification of prostheses effect on gray values of dental CBCT images. Each circular ROI within teeth is composed of 12 pixels. A set of contours outlining ROIs is shown in Fig. 2(b). Figure 2(b) is one slice of the image sets of the 7th experiment. The mean CBCT gray value for each ROI was calculated on images of eight experiments (prosthesis placed in eight dental model casts) and compared with those measured from prosthesis‐free dental cast. CBCT gray values of the prosthesis‐free dental cast were expressed as standard values and expressed in subscript “standard” in Eq. (1). A mean CBCT gray value differences for ith ROI was calculated using following equation:^(23)^

(1) $$Grey\;value\;differences=\frac{\left|Grey-Grey_{\text{Standard}}\right|}{Grey_{\text{Standard}}}\times 100\%$$

“*Grey*” is CBCT gray values for each ROI for all dental CBCT images sets. Gray value differences for each ROI for eight experiments are shown in Table S1 in Appendix A. To compare significance of each design factor on CBCT images, ANOVA^(24)^ assessed the effect of dental prostheses on CBCT images and percent contribution of each factor were calculated. Parameters for ANOVA analysis are total sum of squares (${\text{SS}}_{T}$), factor sum of squares (SS), pure sum of squares (SS), degree of freedom (DOF), variance (V), and percentage of each factor (P).^(25)^ Because gray value differences for each ROI for eight dental model casts is “smaller is better”, the S/N ratio for this type response is:^(26)^

(2) $$SN_{i}=-10{\text{log}}_{10}(\frac{1}{M}\sum_{k=1}^{M}y_{k}{\;}^{2})$$

where $SN_{i}$ is the S/N ratio for ith experiment, *M* is the number of ROIs for calculating S/N ratio, and $y_{k}$ is gray value differences calculated by Eq. (1) for the kth ROI. Total sum of squares term is a measure of deviation of the experimental data from the mean value of the data and can be defined as follows:

(3) $$SS_{T}=\sum_{i=1}^{N}SN_{i}^{2}-CF$$

where *CF* is the correction factor, $CF=\frac{1}{N}(\sum_{i=1}^{N}S{N_{i})}^{2},SN_{i}$ is the S/N ratio for the ith experiment, and *N* is the total number of experiments according to the orthogonal array (N=8 in this work). The factor sum of squares is variation caused by an individual factor. Take factor C, for example; factor sum of squares is given by:

(4) $$SS_{C}=(\frac{C_{1}^{2}}{N_{C1}}+\frac{C_{2}^{2}}{N_{C2}})-CF$$

Total S/N ratio of factor C and number of experiments for factors C due to level 1 and 2 are indicated by $C_{1},\;C_{2}$ and $N_{C1},N_{C2}$. Degree of freedom (DOF) is indication of the amount of information contained in a dataset and DOF is number of levels minus 1. For factor C, the DOF=2−1=1. Mean squares (or variance, V) is the sum of squares per degree of freedom. In this work, since all factors have DOF as 1, variance column in ANOVA shows the same number as total sum of squares term. Pure sum of squares (SS) is the variation caused by an individual factor minus the degree of freedom times the error variance. For factor C, (${\text{SS}}_{C}{\;}^{'}$) is calculated as follows:

(5) $$SS_{C}{\;}^{\prime }=SS_{C}-f_{C}V_{e}$$

where $V_{e}$ is error variance, and $f_{c}$ is DOF of factor *C*. Influence attributed to each significant factor is to find the percentage of individual contributions and is reflected in percent contribution (P) as follows:

(6) $$P=\frac{SS_{C}{\;}^{\prime }}{SS_{T}}\times 100\%$$

The 95% confidence interval of the confirmation experiment is calculated by Eq. (7):

$$CI_{SN}=\sqrt{F_{0.05;1,2}\times V_{e}\times \left(\frac{1}{N_{eff}}\right)}$$

and

(7) $$N_{eff}\frac{N}{1+T_{DOF}}$$

where $F_{0.05;1,2}$ at the confidence level of 95% is 18.51, $N_{eff}$ is effective number of replication of experiment, *N* is total number of experiments (N=8), and $T_{DOF}$ is the total degrees of freedom for ANOVA analysis. All above‐mentioned parameters correspond to ROIs 1–9 and 10–18, and are listed in 3, 4. A detailed description of ANOVA calculation is shown in Appendix B (Eqs. (S1)–(S6)).

**Table 3:** ANOVA results for S/N ratio and gray value differences for ROIs 1–9: factor sum of squares (SS), pure sum of squares (SS′), degree of freedom (DOF), variance (V), percentage of each factor (P), effective number of replication of experiment ($N_{\text{eff}}$) and error variance ($V_{e}$).

*Factor*	*SS*	*DOF*	*V*	*SS′*	*P (%)*
A	0.01	1^a^	0.01		
B	4.15	1^a^	4.15		
C	120	1	120	118	42.7
D	13.5	1	13.5	11.5	4.2
E	54.8	1	54.8	52.8	19
F	13.6	1	13.6	11.6	4.2
G	84.5	1	84.5	82.4	29.8
Total					100

^a^ Error terms in ANOVA analysis.

$F_{0.05;1,2}=18.51$ (F‐ratio at 95% confidence); $V_{e}=({\text{SS}}_{A}+{\text{SS}}_{B})/({\text{DOF}}_{A}+{\text{DPF}}_{B})(\text{error}\;\text{variance});N_{\text{eff}}=8/(1+5)$ (effective number of replication of experiment).

**Table 4:** ANOVA result for S/N ratio and gray value differences for ROIs 10–18.

*Factor*	*SS*	*DOF*	*V*	*SS′*	*P (%)*
A	5.4	1	5.4	4.7	6.2
B	5.3	1	5.3	4.6	6
C	55.7	1	55.7	55	72
D	4.4	1	4.4	3.7	5
E	8.7	1	8.7	8	10.6
F	0.87	1^a^	0.87		
G	0.46	1^a^	0.46		
Total					100

^a^ Error terms in ANOVA analysis.

$F_{0.05;1,2}=18.51$ (F‐ratio at 95% confidence); $V_{e}=({\text{SS}}_{A}+{\text{SS}}_{B})/({\text{DOF}}_{A}+{\text{DPF}}_{B})(\text{error}\;\text{variance});N_{\text{eff}}=8/(1+5)$(effective number of replication of experiment)

## III. RESULTS & DISCUSSION

The Taguchi method can be used to reduce the simulated effort required to investigate multiple factors. The gray value accuracy affected by metal prostheses was analyzed by corresponding S/N ratio of seven factors for eight dental model casts. ANOVA analysis was adopted in discussing significance of seven designed factors.

### A. Gray value differences

**Figure 3:**
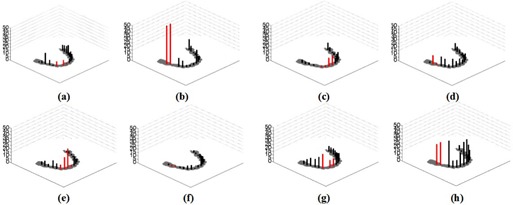
Comparisons of CBCT gray value differences for eight dental casts. Gray value is compared to a prosthesis‐free dental cast; (a) to (h) corresponds to dental casts marked in (a) to (h) in Fig. 1.

Mean gray value differences of eighteen ROIs within four slices of gingiva were analyzed. Figure 3 (a) to (h) shows bar plots of mean CBCT gray value differences for each ROI in the eight dental model casts. Panels (a) to (h) in Fig. 3 correspond to experiments numbered 1–8 in Table 1. According to Table 1, Fig. 3 Panels (a) to (d) were image sets obtained from DCT 100, and Panels e to h were image sets obtained from KaVo eXam. For Panels (c), (d), (g), and (h), the canting of occlusal plane is 15° during scanning. For ROIs containing implants like ROI 7 for 7th dental model cast, the gray value differences are not calculated and there are no bars present S/N ratio of this region. Because gray values of ROIs containing dental implants are definitely high, it is meaningless to compare ROIs containing dental implants like ROI 7 for the 7th experiment with the ROI 7 for prosthesis–free dental model cast. Bars plotted in red are the gray value differences for ROIs next to an implant‐supported prostheses like region 6–9 in Panels (a), (c), (e), (g) and regions 2, 3 in Panels (b), (d), (f), (h). Based on Table S1 in Appendix A, gray value differences for ROIs 1–18 obtained by DCT 100 (experiments 1–4) are 5% less than those obtained by dental CBCT KaVo eXam (experiments 5–8), as shown in Panels (a) to (d) and Panels (e) to (h). One possible reason is that the FOV of the DCT 100 is smaller than the FOV of the KaVo eXam, as reduced FOV provides qualitatively improved image quality.^(27)^ Dental model casts with the condition of implant‐supported prostheses are shown in Panels (a), (b), (g), and (h). It is evident that the gray value differences of regions 2, 3, next to dental implants, were dramatically increased (Panels (b), (g), (h)). Dental model cast with crowns or bridges are shown in Panels (c) to (f). Prosthetic crowns or bridges resulted in better accuracy of gray values (lower gray value differences) compared to those with the support of implants, as shown in Panels (c) to (f). This means that types of restoration have less impact on gray values as compared to implant connection. Gray value differences with the condition of implant‐supported crowns are not significantly large as compared to those with the condition of implant‐supported bridges, as shown in Panels (a) and (g) and Panels (b) and (h). Gray value accuracy increases as distance from dental implants increases. Gray value differences for regions 1–9 were larger than those for regions 10–18. Dental prostheses located at regions 11–18 had minor effects on gray values. For the DCT 100 scanner, gray value differences ranged between 7% and 8% for regions next to single implant‐supported Ti crown (Panel (a)), and 46%–59% for regions between double Ni‐Cr implants (Panel (b)). For 15° dental casts during scanning, gray value differences were between 5% and 8% for regions next to Ti implants, and between 11%–12% for regions under Ni‐Cr alloy prosthetic crowns (see Panels (c) and (d)). For the KaVo eXam scanner, gray value differences were between 16% and 28% for regions next to Ni‐Cr alloy bridges, and between 2% and 5% for regions next to Ti crowns (see Panels (e) and (f)). The gray value differences range from 10% to 20% for regions next to single Ni‐Cr implants with the condition of occlusal plane canting of 15° (see Panel (g)), and 28%–31% for regions between double Ti implants (see Panel (h)). For a single crown prosthesis (without support of implants) placed in dental model casts, gray value differences for ROIs 1–9 were below 12% and gray value differences for ROIs 13–18 away from prostheses were below 10% (see Panels (d) and (f) and Table S1).

### B. Effect of the seven factors and analysis of the S/N ratio

**Figure 4:**
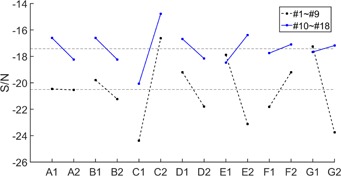
S/N ratio for seven factors (A: CBCT images, B: occlusal plane canting, C: implant connection, D: prosthesis position, E: coping material, F: coping thickness, G: types of dental restoration). The dotted lines represent S/N ratios for ROIs 1–9 and solid lines represent S/N ratio for ROIs 10–18.

Dental CBCT gray values of eight dental models were compared to prosthesis‐free dental model. The S/N ratios for ROIs 1–18 are shown in Fig. 4; the dotted lines represent S/N ratios for ROIs 1–9 and solid lines represent S/N ratio for ROIs 10–18. Greater S/N ratios correspond to better performance. With respect to gray value differences for regions 1–9, it was found that CBCT scanner and occlusal plan canting were not major factors affecting the gray value accuracy of CBCT images, as was denoted as error terms in ANOVA analysis. Gray values were affected much by implant connections. Lower S/N values were found with the condition of implant‐supported prostheses ($S/N_{C1}<S/N_{C2}$). Gray value differences were better with the condition of anterior prostheses as compared to the conditions with posterior prostheses ($S/N_{D1}>S/N_{D2}$). This observation explains Kacer's work,^(27)^ in that the survival rate for loaded implants in the anterior mandible is higher than those for the survival rate in the posterior mandible. The more correct prediction of bone condition is with the condition of anterior mandible than with the condition of posterior mandible. The S/N ratio was better with the condition of Ti prosthesis than with the condition of Ni‐Cr prosthesis ($S/N_{E1}>S/N_{E2}$). The accuracy of gray values of CBCT was lower with thinner coping thickness ($S/N_{F1}<S/N_{F2}$). Performance was better with the condition of prosthetic crown as compared to prosthetic bridge ($S/N_{G1}>S/N_{G2}$). Implant connection was a major factor affecting the gray value accuracy of maxillary gingiva (42%), followed by type of restoration (29%), prosthesis position (19%), coping material (4%), and coping thickness (4%) (see Table 3). Accuracy of CBCT gray values was worst with the condition of implant‐supported posterior Ni‐Cr bridges with a coping thickness of 3 mm ($S/N_{C1,D2,E2,F1,G2}$). The main factors effecting gray value were implant connection ((p=0.012)) and type of restoration ((p=0.016)). The p‐value was obtained by ANOVA command in MATLAB. A value of *p* less than 0.05 indicated a statistically significant difference. Occusal plan canting and CBCT scanners were not major factors affecting gray value differences of gingiva for ROIs 1–9. A confirmation test was used to verify the results based on Taguchi's design approach. The upper and lower limits of estimated performance at the optimum condition are expected result $\pm {\text{CI}}_{\text{SN}}$. The S/N ratio for gray value differences was −8.15 with optimal setting of dental prosthesis. A verification experiment was conducted by the 6th dental model cast and gray value differences were found to be −10.07. This result was within the limit for the range of estimated performance at the optimum condition ($-8.15\;\pm {\text{CI}}_{\text{SN}}$).

With respect to ROIs 10–18, the S/N value of seven factors for regions 10–18 (Fig. 4, solid lines) were better compared to that for regions 1–9 (Fig. 4, dotted lines). The contributions of the six factors to accuracy of gray value were similar, except for the factor‐implant connection. For ROIs 10–18, implant connection was a major factor affecting the gray value accuracy of maxillary gingiva (72%), followed by coping material (10%), and the other factors (6%) (Table 4). Coping thickness and types of restoration were denoted as error terms in ANOVA analysis. Difference of CBCT gray values obtained by the DCT 100 were better than those from the KaVo eXam CBCT scanner ($S/N_{A1}>S/N_{A2}$). Performance was better with the condition of occlusal plan canting of 0° than with condition of 15° ($S/N_{B1}>S/N_{B2}$). Significant differences were found in the condition of implant‐supported prostheses ($S/N_{C1}<S/N_{C2}$). Accuracy was better with the condition of anterior prosthesis as compared to conditions with posterior prosthesis ($S/N_{D1}>S/N_{D2}$). Gray value accuracy was better with the condition of Ni‐Cr prosthesis than with the condition of Ti prosthesis ($S/N_{E1}<S/N_{E2}$).

## IV. CONCLUSIONS

Systematic dental cast designs based on the Taguchi method were applied to analyze the effect of dental prostheses on CBCT images. Eight experiments were based on an $L_{8}$ orthogonal array. The 95% confidence intervals of the confirmation experiment verified the results. The mean gray value of regions within gingiva in the vicinity of dental prosthesis was significantly affected, particularly in the area between implant‐supported prostheses. For implant‐supported prosthesis, gray value differences for regions next to implant‐supported prostheses were dramatically increased. The largest gray value difference was 59% for regions between double implants‐supported Ni‐Cr prosthesis (ROIs 2, 3 for 2nd experiment), and the smallest gray value difference was 7% for regions next to single implant‐supported Ti prosthesis (ROIs 6, 8 for 1st experiment). For a single crown prosthesis (without support of implants) placed in dental model casts, gray value differences for ROIs 1–9 were below 12% and gray value differences for ROIs 13–18 away from prostheses were below 10%. The dental CBCT gray value was influenced mainly by implant connection (42%), followed by type of restoration (29%), prosthesis position (19%), prosthesis material (4%), and coping thickness (4%). Scanners and occlusal plane canting were not major factors affecting the gray values of regions surrounding dental prostheses. Overall, this study has shown the effect of scanning parameters and dental prostheses on dental CBCT gray values. We suggest that the range of gray value variation for regions next to dental prostheses be based on a systematic experiment. The variation of gray values for ROIs within gingiva in the vicinity of dental prosthesis was pointed out, and we hope that suggested gray value difference affected by prostheses is an indicator for clinical diagnosis of bone quality.

## ACKNOWLEDGMENTS

We would like to thank Taiwan CareTech Corporation for their assistance and facilities which allowed this research.

## Supporting information

Supplementary Material

Supplementary Material

Supplementary Material

## Appendix A. Gray Value Differences for the 18 ROIs.

**Table S1:** Gray value differences for all 18 ROIs. For ROIs containing implants, like ROI 7 for 7th dental model cast, the gray value differences are not calculated.

*Exp. No/ROIs*	*1*	*2*	*3*	*4*	*5*	*6*	7	*8*	*9*	*10*	*11*	*12*	*13*	*14*	*15*	*16*	*17*	*18*	*S/N ratio for ROIs 1‐9*	*S/N ratio for ROIs 10‐18*
1	0.87	4.14	16.89	7.43	0.88	7.21		8.01	1.56	6.00	5.25	8.14	10.39	5.80	14.98	10.93	9.42	7.91	−17.76	−19.30
2		46.17	59.18		16.58	7.81	15.34	2.49	3.21	9.02	2.84	5.24	5.68	8.68	0.02	2.12	7.09	15.29	−29.49	−17.56
3	8.31	0.46	6.08	4.86	1.90	5.33	8.32	6.72	5.59	6.94	9.39	7.61	2.97	1.06	3.86	0.12	4.18	4.19	−15.34	−14.51
4	5.89	11.03	3.03	12.82	11.75	3.45	9.88	7.87	11.19	7.52	7.24	7.82	3.25	4.19	0.90	5.68	4.38	6.12	−19.29	−15.05
5	4.78	7.39	3.96	10.31	7.27	6.27	16.65	28.14	2.65	10.28	4.91	4.36	2.50	3.76	1.28	1.23	3.22	0.97	−21.82	−13.10
6	1.82	1.63	1.45	2.12	2.79	5.46	5.84	0.55	2.62	13.72	9.50	4.49	6.87	4.78	4.74	0.79	1.38	2.73	−10.07	−16.49
7	4.57	8.36	11.09	11.49	15.53	20.52		10.65	10.83	11.93	13.23	11.25	9.87	8.42	8.56	8.43	7.15	7.45	−21.90	−19.82
8		28.13	31.98		36.93	9.67	10.01	19.73	32.58	34.56	24.38	14.21	3.29	0.38	0.31	1.04	1.29	6.48	−28.38	−23.57

## Appendix B. Description of ANOVA Calculation.

ANOVA calculation for ROIs 1‐9 is as follows:

(B.1) $$SN_{i}=-10{\text{log}}_{10}(\frac{1}{M}\sum_{k=1}^{M}y_{k}{\;}^{2})$$

For the 1st experiment, the term $\frac{1}{M}\sum_{i=1}^{M}{y_{i}}^{2}$ is calculated as follows:

(B.2) $$\begin{array}{l} \frac{1}{M}\sum_{k=1}^{M}y_{k}{\;}^{2}\\ =\frac{1}{8}(0.87^{2}+4.14^{2}+16.89^{2}+7.43^{2}+0.88^{2}+7.21^{2}+8.01^{2}+1.56^{2})=59.7 \end{array}$$

Total sum of squares term is given by:

(B.3) $$\begin{array}{l} SS_{T}\\ =\sum_{i=1}^{N}SN_{i}^{2}-CF\\ =({\left(-17.76\right)}^{2}+{\left(-29.48\right)}^{2}+\ldots +{\left(-28.38\right)}^{2})-\frac{{\left(-17.76-29.48-\ldots -28.38\right)}^{2}}{8}=291 \end{array}$$

The factor sum of squares for factor C is given by:

(B.4) $$\begin{array}{l} SS_{C}\\ (\frac{C_{1}^{2}}{N_{C1}}+\frac{C_{2}^{2}}{N_{C2}})-CF\\ =\frac{1}{4}({\left(-17.76-29.48-21.89-28.38\right)}^{2}+{\left(-15.33-19.29-21.81-10.06\right)}^{2})\\ =\frac{{\left(-17.76-29.48-\ldots -28.38\right)}^{2}}{8}\\ =0.01 \end{array}$$

Total S/N ratio due to level 1 and 2 of factor A are indicated by $C_{1,2}$, and $N_{C1,C2}$ are number of experiment due to factor A with levels 1, 2. Because factor sum of squares of factor A and B (${\text{SS}}_{A},\;{\text{SS}}_{B}$) are much lower than those of other factors, these two terms were denoted as error terms in ANOVA analysis. Pure sum of squares is given by:

(B.5) $$SS_{C}{\;}^{\prime }=SS_{C}-f_{C}V_{e}$$

where $f_{c}$ is DOF for factor *C* and $V_{e}$ is error variance and is given by:

(B.6) $$V_{e}=\frac{\left(SS_{A}+SS_{B}\right)}{2}$$

The confidence interval (${\text{CI}}_{\text{SN}}$) represents the boundaries on the expected results and is always calculated at a 95% confidence level.

(B.7) $$\begin{array}{l} CI_{SN}=\sqrt[{\pm }]{F_{0.05;1,2}\times V_{e}\times \left(\frac{1}{N_{eff}}\right)}=\sqrt[{\pm }]{18.51\times (\frac{SS_{A}+SS_{B}}{2})\times \left(\frac{1}{N_{eff}}\right)}\\ =\pm 5.38\\ N_{eff}=\frac{N}{1+T_{DOF}} \end{array}$$

where $F_{\alpha ;1,fe}(F_{0.05;1,2})$ is the F–ratio required for 100(1−α) percent confidence interval, *fe* is DOF for error, $V_{e}$ is the error variance, $N_{eff}$ is effective number of replication of experiment, *N* is total number of experiments (N=8) and $T_{DOF}$ is the total degrees of freedom for ANOVA analysis ($T_{\text{DOF}}=5$). The upper and lower limits of estimated performance at the optimum condition are expected result $\pm {\text{CI}}_{\text{SN}}$. The S/N ratio for gray value differences was −8.15 with optimal setting of dental prosthesis. A verification experiment was conducted by the 6th dental model cast and gray value differences were found to be −10.07. This result was within the limit for the range of estimated performance at the optimum condition ($-8.15\;\pm {\text{CI}}_{\text{SN}}$).
