# Supplementary material for: Quantification of dental prostheses on cone‐beam CT images by the Taguchi method
Source: J Appl Clin Med Phys. 2016 Jan 8;17(1):207–20. doi: 10.1120/jacmp.v17i1.5826 (PMC5690196; doi:10.1120/jacmp.v17i1.5826)
Supplement: Supplementary file 2 — Supplementary Material [file ACM2-17-207-s002.docx]

**Supporting Information**

Table S1 Grey value differences for 18 ROIs. For ROIs containing implants like ROI 7 for 7th dental model cast, the grey value differences are not calculated.

ANOVA calculation for ROIs 1-9 is as follows

$SN_{i}=-10\log_{10}(\frac{1}{M}\sum_{k=1}^{M} y_{k}^{2})$ (S1)

For the 1st experiment, the term $\frac{1}{M}\sum_{k=1}^{M} y_{k}^{2}$ is calculated as follows:

$\begin{matrix} \frac{1}{M}\sum_{k=1}^{M} y_{k}^{2} \\ =\frac{1}{8}(0.87^{2}+\text{4.14 }^{2}+16.89^{2}\text{ }+7.43^{2}\text{ }+0.88^{2}+7.21^{2}+8.01^{2}+1.56^{2})\text{ }=59.7 \end{matrix}$ (S2)

Total sum of squares term is given by

$\begin{matrix} SS_{T} \\ =\sum_{i=1}^{N} SN_{i}^{2}-CF\text{ } \\ =((\text{-17.76)}^{2}+\text{(-29.48)}^{2}+...+\text{(-28.38)}^{2}\text{)-}\frac{\text{(-17.76-29.48-...-28.38)}^{2}}{8}=291 \end{matrix}$ (S3)

The factor sum of squares for factor C is given by

$\begin{matrix} SS_{C} \\ =(\frac{C_{1}^{2}}{N_{C1}}+\frac{C_{2}^{2}}{N_{C2}})-CF \\ =\frac{1}{4}((\text{-17.76-29.48-21.89-28.38)}^{2}+(\text{-15.33-19.29-21.81-10.06})^{2}) \\ -\frac{\text{(-17.76-29.48-...-28.38)}^{2}}{8} \\ =0.01 \end{matrix}$ (S4)

Total S/N ratio due to level 1 and 2 of factor A are indicated by C_1_, _2_, N_C1,C2_ are number of experiment due to factor A with levels 1, 2. Because factor sum of squares of factor A and B (SS_A_, SS_B_) are much lower than those of other factors, these two terms were denoted as error terms in ANOVA analysis. Pure sum of squares is given by

$SS_{C}'=SS_{C}-f_{C}V_{\text{e}}$ (S5)

Where fc is DOF for factor C and *V*_e_ is given by

$$V_{e}=\frac{\left(SS_{A}+SS_{B}\right)}{2}$$

The confidence interval (CI_SN_) represents the boundaries on the expected results and is always calculated at a 95% confidence level.

$\begin{matrix} CI_{SN}=\pm\sqrt{F_{0.05;1,2}\times V_{e}\times\left(\frac{1}{N_{eff}}\right)}=\pm\sqrt{18.51\times(\frac{SS_{A}+SS_{B}}{2})\times\left(\frac{1}{N_{eff}}\right)} \\ =\pm5.38 \end{matrix}$ (S6)

$$\text{N}_{\begin{matrix} eff \\ \end{matrix}}=\frac{N}{1+T_{DOF}}$$

N_eff_ is effective number of replication of experiment, N is total number of experiments (N = 8) and T_DOF_ is the total degrees of freedom for ANOVA analysis (T_DOF_=5). The upper and lower limits of estimated performance at the optimum condition are expected result ±CI_SN_. The S/N ratio for grey value differences was -8.15 with optimal setting of dental prosthesis. A verification experiment was conducted by the 6th dental model cast and grey value differences was found to be -10.07. This result was within the limit for the range of estimated performance at the optimum condition (-8.15 ± CI_SN_).
